# Co-producing Human and Animal Experimental Subjects: Exploring the Views of UK COVID-19 Vaccine Trial Participants on Animal Testing

**DOI:** 10.1177/01622439211057084

**Published:** 2021-11-15

**Authors:** Samantha Vanderslott, Alexandra Palmer, Tonia Thomas, Beth Greenhough, Arabella Stuart, John A. Henry, Marcus English, Rebecca de Water Naude, Maia Patrick-Smith, Naomi Douglas, Maria Moore, Susanne H. Hodgson, Katherine R. W. Emary, Andrew J. Pollard

**Affiliations:** 1 Oxford Vaccine Group, Department of Paediatrics, NIHR Oxford Biomedical Research Centre, University of Oxford, United Kingdom; 2 Oxford Martin School, University of Oxford, United Kingdom; 3 School of Geography and the Environment, Oxford University Centre for the Environment, University of Oxford, United Kingdom; 4 Medical Sciences Division, University of Oxford Medical School, University of Oxford, United Kingdom; 5 Jenner Institute, Nuffield Department of Medicine, University of Oxford, United Kingdom

**Keywords:** COVID-19, vaccine, animal research, clinical trial, co-production

## Abstract

Preclinical (animal) testing and human testing of drugs and vaccines are rarely considered by social scientists side by side. Where this is done, it is typically for theoretically exploring the ethics of the two situations to compare relative treatment. In contrast, we empirically explore how human clinical trial participants understand the role of animal test subjects in vaccine development. Furthermore, social science research has only concentrated on broad public opinion and the views of patients about animal research, whereas we explore the views of a public group particularly implicated in pharmaceutical development: *experimental subjects*. We surveyed and interviewed COVID-19 vaccine trial participants in Oxford, UK, on their views about taking part in a vaccine trial and the role of animals in trials. We found that trial participants mirrored assumptions about legitimate reasons for animal testing embedded in regulation and provided insight into (i) the nuances of public opinion on animal research; (ii) the co-production of human and animal experimental subjects; (iii) how vaccine and medicine testing, and the motivations and demographics of clinical trial participants, change in an outbreak; and (iv) what public involvement can offer to science.

## Introduction

New pharmaceutical products, including vaccines, are first tested in nonhuman animals (hereafter, animals) for safety. Because animal and human biology are not identical, medicines must then be tested on humans before regulatory approval. A large amount has been written about the social and ethical dimensions of both elements of animal and human testing, including the motivations and experiences of healthy clinical trial participants in non-outbreak scenarios (Abadie 2010; Fisher 2020; Mwale 2017; Petryna 2009; Rajan 2005; Tengbeh et al. 2018) and how scientists, laboratory workers, and regulators think about the ethics, practices, and public perceptions of animal testing (Davies et al. 2020; Hobson-West 2010; Hobson-West and Davies 2018; Sharp 2019).

Rarely, however, are these human and nonhuman elements of testing considered side by side. Where this is done, it typically involves examining clinical trial participants’ use of “guinea pig” and “lab rat” metaphors (Abadie 2010; Fisher 2020) or theoretically exploring the ethics of these two situations to compare relative treatment. For example, animal testing has been compared favorably to the recruitment of healthy clinical trial participants in the United States (US) given the exploitative treatment of phase I clinical trial participants in some contexts (Fisher and Walker 2019). Discussions have also focused on the difference between the need for informed consent from human research participants compared with the lack of choice given to animals and how “assent” from research animals might be secured (Palmer et al. forthcoming; Mancini 2017). Such discussions echo conversations about how to undertake ethical research with children (MRC 2004), and how to ensure that veterinary treatments are in the best interests of animals rather than owners (Ashall, Millar, and Hobson-West 2018).

“Co-production” is an idiom first described by Jasanoff (2004) for “how the orderings of nature and society reinforce each other” (p. 17). Applied to animal research, co-production emphasizes removing the boundary between “the natural” of animals and science, and “the social” of political power, culture, and public views. Viewing humans and animals as engaged in co-production involves acknowledging that it is not only humans that have agency and influence animals but also the reverse: animals are agents that influence our attitudes, behaviors, identities, and well-being and the production of scientific knowledge. For example, social scientists have examined how humans and animals co-produce health and well-being in care farms, where people with learning difficulties or mental illness spend time caring for animals (often themselves undergoing rehabilitation) as a therapeutic intervention (Gorman and Cacciatore 2017). In the context of animal research, scholars have explored co-production through of patient and public involvement (PPI), which aims to improve the quality and relevance of animal research by incorporating patients’ priorities and drawing on their lived experiences, for example, by inviting patients to visit animal research facilities or involving them in research review. As Gorman and Davies (2020) observe, such practices offer opportunities for patients and the public to connect care for humans and animals.

Thus, co-production of health and care has been examined in relation to research animals and patient groups. However, to our knowledge, social scientists have not explored the co-production of human and animal *experimental subjects*: humans and animals whose bodies are sites for testing, but who may not directly benefit from the product being tested. Through exploring COVID-19 vaccine clinical trial participants’ views on animal testing, we explore how their identities as test subjects are produced in relation to research animals. In short, we unpack *how human and animal experimental identities are co-produced*. We propose that doing so can inform understanding of the ethics and practices of experimentation and provide insight into interspecies subjectivities and identities through a reflection on humans’ perceptions of their animal counterparts.

Our analysis also contributes to the important conversations about the role of public groups in steering the direction of animal research. Animal testing has long been a subject of public interest. In the UK, debate has been ongoing since at least the nineteenth century, though the nature of discussions has changed over time (Davies et al. 2020). Public opinion is viewed as important for both advocates and opponents of animal research, with both “sides” commonly citing opinion polls as justification for their stance–the goal being to convey rationality and moral and democratic legitimacy (Hobson-West 2010). Regulators also acknowledge shaping guidance and practices in response to what they think represents the public view, for example, devoting the greatest oversight to species (e.g., primates, companion animals) and practices presumed to be the least publicly accepted (Hobson-West and Davies 2018).

In light of these concerns, various efforts are made to bring public groups, such as patients, on board. Regulation is one such approach, with the UK’s Animals (Scientific Procedures) Act of 1986 drafted with the aim of reaching a compromise between the interests and concerns of scientists, vets and other animal welfare advocates, and the public (Myelnikov 2019). In recent years, transparency has emerged as another strategy for building better relationships between public and animal research communities, such as via the Concordat on Openness launched in 2014 by a coalition of UK animal research groups. This move is founded on the belief that openness is essential for positive science–society relationships by moving away from a deficit model, in which public opposition to science is assumed to be a product of insufficient knowledge, toward more socially accountable “mode 2” science (McLeod and Hobson-West 2016). In this paper, we add important empirical detail to these conversations by describing the views of a particularly important public on animal testing.

We begin by describing how the ChAdOx1 nCoV-19 vaccine (ClinicalTrials.gov identifier: NCT04324606), commonly known as the Oxford or AstraZeneca vaccine, was tested on animals and humans; and how animal testing was presented to volunteers and public groups. We then explore human volunteers’ perspectives on whether animal testing is necessary, before examining how the trial participants spoke about their own roles as experimental subjects in relation to animals. We conclude by reflecting on what these findings tell us about public opinion on animal research, the co-production of human and animal experimental subjects, how vaccine and medicine testing change in an outbreak, and what public involvement can offer to science.

## Early Human and Animal Testing of ChAdOx nCoV-19

**Figure 1.**
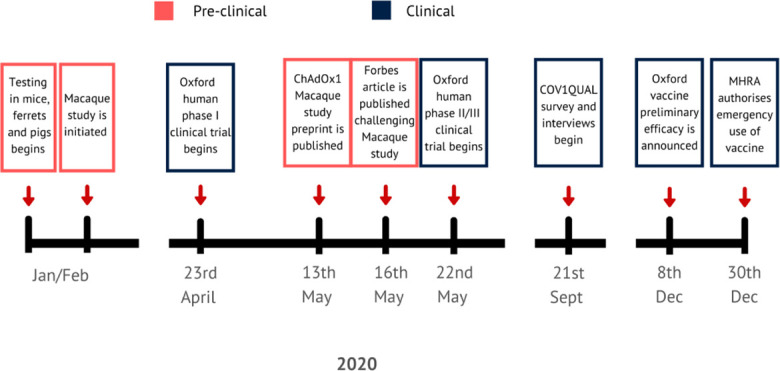
Time line showing key moments in the testing of the ChAdOx nCoV-19 vaccine.

Under normal circumstances, key safety and toxicology studies, and sometimes efficacy studies, are completed before human trials start, mainly because successful animal trials are necessary to secure funding and regulatory approval for human trials. Given the time imperative for development of a COVID-19 vaccine, animal testing of ChAdOx nCoV-19 started extremely quickly and there were only days between the availability of safety and efficacy data from studies in macaques and administration of the first dose in humans (Figure 1).

The vaccine underwent testing in ferrets, mice, and non-human primates at the Public Health England (PHE) laboratories as well as in pigs via collaboration with researchers at the Pirbright Institute (MRC 2021). Further assessment in rhesus macaques (selected because they are considered to be genetically closer to humans) in the US showed that six vaccinated macaques had no pathological changes and a reduced viral load in lung secretions compared with three unvaccinated macaques, but there was no difference in viral shedding in nasal secretions between the groups (Van Doremalen et al. 2020). The results generated considerable publicity, prompting speculation about the vaccine’s efficacy (e.g., in *Forbes*: Haseltine 2020). Other questions raised in public centered on the ethics and practice of accelerating, performing in parallel, or even removing animal testing from vaccine development in a pandemic.

Animal testing featured in the participant information sheet (PIS) for the study (see Online Supplement) and corresponding video. The PIS stated, “Until now, this vaccine has only been tested on laboratory mice and other animal species and this is the first time that the vaccine will be given to humans” (COVID-19 Oxford Vaccine Trial 2021). The PIS also referenced prior mouse studies of vaccines against a related coronavirus, the SARS virus, by different research groups, in which vaccinated animals developed more severe lung inflammation on infection than unvaccinated animals. Given that the COVID-19 virus is in the same family as the SARS virus and also infects the lungs, there was a theoretical risk that participants could develop enhanced COVID-19 disease due to vaccination, although this proved not to be the case. The trial website Q&A also referenced testing in animals, for example, the finding of good safety and efficacy from the macaque studies (COVID-19 Oxford Vaccine Trial 2021). Data presented to participants were reviewed by an independent data and safety monitoring board, as well as ethical and regulatory review, before the trial.

### Survey Methodology and Data Analysis

The first clinical trial in humans of ChAdOx nCoV-19 started in April 2020 as a single-blinded, randomized phase I/II trial (COV001; Figure 1). The trial enrolled more than 1,000 healthy participants aged 18 to 55 across five UK study sites in Oxford, Southampton, Bristol, and London, to assess the safety of and immune response to the vaccine. From this group, we invited 770 participants screened in Oxford who had indicated they were happy to be contacted about other studies to participate in COVQUAL, a mixed methods study exploring COVID-19 vaccine trial participants’ motivations for and experiences in participating in COV001. Three hundred forty-nine participants completed an online survey, 102 of whom also participated in a semi-structured interview. COVQUAL was approved by the University of Oxford Central University Research Ethics Committee (ref: R70147_CUREC), and informed consent was obtained in writing for both the survey and interviews and additionally orally in interview recordings.

Survey respondents were quite evenly split by gender (55 percent identified as female), and 45 to 55 years was the largest age-group (33 percent, 114 of 349). Most respondents reported their nationality as British, (84 percent, 292 of 349), 8 percent (28 of 349) reported being European, and 2 percent (8 of 349) American. Other nationalities included New Zealand, Mexican, Filipino, Canadian, and Japanese. Most respondents identified as white British (77 percent, 267 of 349), with only 6 percent (21 of 349) describing their ethnicity as Black, Asian, and Minority Ethnic (BAME). More than half of respondents (56 percent, 194 of 349) were educated to postgraduate level and 56 percent (197 of 349) were employed full time, with education, law, and government services the most commonly reported occupational groups. About 40 percent (138 of 349) were living with a partner, 50 percent (173 of 349) were single, and 62 percent (216 of 349) were without children (see Online Supplement for further demographic information).

Interviews took place via Microsoft Teams and were recorded and transcribed verbatim. Interviews typically took between forty-five minutes to one and half hours and were conducted by eleven members of the study team. We believe that the survey response was high because this appeared to be a group highly motivated to be involved in research, with a view that the trial (and associated research) was of high importance. The pandemic meant that many were working from home or furloughed, which offered some flexibility in being able to take part in interviews conducted remotely (several interviewees commented on this).

We conducted a thematic and discourse analysis of the interview transcripts using NVivo version 12, in order to identify key themes and patterns occurring in discussions, as well as connections between subtopics to link types of conversation clusters together. A codebook was developed iteratively by the team, and regular meetings were used to check for coding consistency. We also conducted an inter-coder reliability test that demonstrated good-to-excellent reliability. Such an approach draws on Lewis, Hughes, and Atkinson (2014), where empirical materials are used as part of an iterative process of analysis. We additionally analyzed survey data using descriptive statistics to identify key trends.

This paper draws predominantly on our analysis of interview material where animal testing is discussed. We also selectively use the survey data on demographics as well as two relevant questions about the motivation to take part in the trial and the risk posed by the quick turnaround from animal to human testing. We do not use the interview and survey data in full because they incorporated a wide range of topics, including opinions about the general risk and safety of the trial, media representation, experience of taking part in the trial during the pandemic, and attitudes to vaccines. This information is not relevant to the research questions of the current paper and were therefore not included. The full survey questionnaire and interview guide are contained in the Online Supplement.

### Motivations to Participate

The demographic profile could help explain why financial compensation was cited as an important motivator by only 4 percent (13 of 349) of survey respondents. More commonly, people spoke of being motivated by a desire to “help” or being personally affected by COVID-19. One participant (CQS-65481067), for example, explained, “I lost a family member to COVID shortly before my vaccination and would not wish the experience of losing someone during this pandemic on anyone.” Respondents also referred to other altruistic actions like blood donation in explaining why they participated. As one put it:

It’s a bit like blood or organ donation: potential huge gain to someone else for little or no cost to myself, so there’s not really any reason not to. In normal times this wouldn’t apply, since it would have had a bigger impact on everyday life. (CQS-65490989)

Others also referred to the uniqueness of the COVID-19 outbreak and lockdown as important for encouraging their involvement, with one respondent (CQS-65481676) explaining, “It was something fun to do—we were in lockdown and there was very little else!”

Participants for phase I clinical trials of the ChAdOx nCoV-19 vaccine were typically employed professionals educated to degree level. This stands in contrast with phase I clinical trials in the US, which often attract economically disadvantaged, ethnic minority men (Abadie 2010; Fisher 2020; Fisher and Walker 2019), and in resource-poor countries where participants may lack employment or ready access to medicines (Petryna 2009; Rajan 2005). In the UK, the situation is more complex, with paid clinical trials attracting people from a variety of backgrounds and income levels, though financial compensation still tends to be a primary motivation for participation (Mwale 2017). Perhaps, then, the context of the COVID-19 crisis served to make phase I vaccine trial participation in the UK closer to scenarios like biobank and blood donation, in which people often explain their motivations in terms of altruism and progressing science (Hoeyer 2003; Parry and Greenhough 2018).

Some participants raised the subject of animal testing during interviews spontaneously. Where this didn’t happen, we asked them: *Is animal testing necessary to develop vaccines and treatments?* We examine people’s answers in relation to their views on (i) justifications for animal testing and (ii) the relationships between human and animal experimental subjects.

## Justifying Animal Testing

### A Necessary Evil?

Participants repeatedly raised the idea that animal testing is a “necessary evil” (CQI-0343). For example, one participant reflected that “for vaccines and medical trials where it’s potentially for the use of something that’s going to cure or help humans, I think sometimes animal testing is unavoidable” (CQI-0477). The same participant argued, speaking specifically about vaccines, “we have to be sure that they’re not going to kill people.” Another proposed that humans and animals have equal moral worth, meaning that animal testing can be simultaneously wrong and necessary:

I don’t think it’s right, but I think it is necessary. … Yes, I do firmly believe that life is very important no matter what you are. Whether you are a chicken or you are a person, but I feel for the sake of mankind, it’s necessary to test on animals first, yes. (CQI-0337)

In other words, participants generally supported the use of animals in testing for vaccines and medicines, reasoning that animal lives are worth compromising or sacrificing for the sake of saving humans. This reasoning closely aligns with the ethics adopted in the UK’s Animals (Scientific Procedures) Act (A(SP)A). Embedded in A(SP)A is an ethic of utilitarianism, exemplified most clearly by the obligation of Home Office inspectors tasked with approving research, and local Animal Welfare and Ethical Review Bodies (AWERBs; RSPCA and LASA 2015), to weigh up the anticipated harms and benefits of research (Animals in Science Committee 2017). Thus, regulation allows for harm to animals where the benefits are sufficiently justified. In terms of justifications, medical research where there are no alternatives typically emerges in opinion polls as the most widely accepted reason for animal testing (Ipsos MORI 2018).

UK regulation also heavily emphasizes humaneness and minimizing animal suffering, for example, via its emphasis on the 3Rs: the commitment to *Reduce* the number of animals used, *Refine* methods so as to minimize harm, and *Replace* animal research with alternatives whenever possible (Davies et al. 2018). Echoing this sentiment, participants at times spontaneously raised the importance of humaneness: “if it’s a matter of life and death, then I would be more inclined to say, in that case, please test humanely” (CQI-0354). The necessity or not of animal testing was also linked to the need for yet-to-be developed alternatives, with participants qualifying their responses with comments such as “I wish there were other ways to do such things” (CQI-0477) and “the sooner we can find a better way of doing something different fantastic” (CQI-0343).

### Cosmetic Testing in the Public Imaginary

Several participants responded to the question by drawing comparisons with other forms of animal use, for example, by referring to cosmetic testing as an unjustified reason for testing on animals:

…I think things like testing cosmetics on animals, I’d be totally against because I don’t feel there’s any need for me to have lipstick that’s been tested on rabbits. Why should I have nice lipstick and a rabbit is harmed in order to give me plumper lips or whatever? That seems to be ridiculous. (CQI-0329)

Opinion polls typically show that a substantial minority (38 percent in 2018) believe that testing cosmetics on animals is permitted in the UK (Ipsos MORI 2018), despite the fact that this has been banned in the European Union (EU, including the UK) since 1998 (UAR 2020). Cosmetic testing on animals has played an important role in opposition to animal testing in general. For example, in the US in the 1970s and 1980s, activist Henry Spira famously campaigned for cosmetics companies, especially Revlon, to cease their use of the “Draize test,” which involved restraining rabbits and dropping chemical substances into their eyes as a way of testing for toxicity (Weisskircher 2019). Furthermore, it remains alive in public discourses via the anti-cosmetic testing stance still prominently advertised by brands such as Lush, which construct opposition to cosmetic testing on animals as a central element of ethical consumption practices (Aronczyk 2013). Participants’ responses suggest that cosmetic testing continues to serve as an important touchpoint in the public imaginary (i.e., a widespread set of values and symbols) as an example of “bad” animal testing.

### Dietary Choices as Ethical Touchpoints

Two participants spoke of cosmetic testing alongside references to vegetarianism and veganism. As another area in which companies (e.g., those producing plant milks and alternative proteins) directly appeal to consumers via narratives of ethical or responsible consumption, dietary ethics served as another touchpoint for participants seeking to articulate their views about the ethics of animal testing. This offers an interesting parallel with the “ethical boundary-work” (i.e., defense of a practice via reference to another domain) undertaken by scientists who conduct animal research. Scientists in this context complain of being unfairly singled out by animal advocates, on the grounds that their work is more humane, and better regulated and justified, compared with other domains of animal use such as farming (Hobson-West 2012). In a sense, participants also undertook ethical boundary-work by seeking to compare the ethics of food consumption with animal testing. For example, one self-identified vegetarian made the case that if you accept any consumption of animal products whatsoever, you should accept the need for humane and well-justified animal testing:

Unless you live in a fully vegan lifestyle, I don’t think you have any right saying that it shouldn’t be tested on animals. … Because if you’re willing to eat an animal, if you’re willing to wear its skin, if you’re willing to drink milk or eat butter and stuff, then what’s the difference? In terms of what the point is, it’s to sustain life. And I think that all that can really be asked is that the animals were treated with care as much as reasonably possible. (CQI-0334)

Interestingly, a similar point was made by a participant (CQI-0306) who referred to their identity as a meat-eater: “I don’t really feel like I can comment on animal testing if I eat meat.” Thus, even though one participant was vegetarian and the other a meat-eater, both argued that their choices in the domain of food consumption mean that they cannot reasonably object to animal testing.

In sum, participants’ perspectives on the justifications for animal testing suggest broad alignment with the principles embedded in UK animal research regulation and with the views often expressed by researchers and laboratory workers (Greenhough and Roe 2018; Sharp 2019). This did not, however, prevent people from expressing uneasiness about animal testing—“I can understand why they do it, but it makes me uncomfortable” (CQI-0479)—or from acknowledging that answering questions about when and why animal testing is justified is “a difficult one” (CQI-0334). Public groups involved in the ChAdOx nCoV-19 vaccine trial were therefore attuned to the complexities and difficulties of animal testing.

### Expertise and Trust

Despite their nuanced perspectives on the ethics of animal testing, participants often caveated their statements by noting that they are not experts, again echoing the findings of a recent poll in which 38 percent of those surveyed felt “not very well informed” and a further 26 percent “not at all informed” about animal research (Ipsos MORI 2018). Six noted that they did not think they were informed enough to give a clear answer or provided an answer but noted lack of expertise, for example:

I don’t know enough about the science. … People are trying to reduce animal experiments to the minimum, so I assume the fact that they still do it means it’s necessary. (CQI-0341)

I’m not an expert. I don’t know.…I can’t give an easy yes or no answer, because I don’t think I know enough. But again, I have to trust that there are people that know and that they’re doing it in the best way that they can, I guess. (CQI-0472)

Conversely, one participant attributed their certainty that animal testing is necessary to their role as a health-care professional: “I’m from healthcare, I know that you need to test on animals first, so that actually reassured me more than anything, that they had done that” (CQI-0436). Both self-identified experts and nonexperts therefore saw the scientific merit of research as relevant for assessing ethics. This idea is also embedded in the UK’s animal research regulations, with A(SP)A license applications requiring thorough justification of the scientific merits of the proposed research and where possible evidence of peer review (e.g., of funding).

Despite acknowledging the relevance of science for making ethical judgments, and their own lack of scientific expertise, participants also expressed trust that someone more qualified has already determined that animal testing is necessary as captured by use of phrases like “I assume.” This trust in authority may, however, be more widespread among these clinical trial participants than the broader public. According to the 2011 Census, 43 percent of Oxford’s population was qualified to degree level or above, compared with the England average of 27 percent. Clinical participants followed this trend. Recent surveys demonstrate the heightened trust in science that comes with education, particularly in science subjects (Wellcome Global Monitor 2018).

This general sense of trust was captured in responses to survey questions about the vaccine’s risks and likelihood of success: while over a third (130 of 349, 37 percent) thought that new vaccines are riskier than established ones, half (175 of 349, 50 percent) thought that the vaccine would work. Indeed, clinical trial participants almost by definition would not include vaccine critics given their willingness to facilitate vaccine production and thus may be more broadly trusting of scientists than the average Briton. However, one interviewee presented a slightly different perspective, arguing that vaccines have only become necessary due to modern (and undesirable) living conditions. They connected this view to their opposition to animal testing:

the reason it seems to me that we have to have vaccinations is because viruses can now, and other diseases, can spread so easily through a heavily urbanized, globally connected population … So, it’s because of the world we’ve made that vaccines become necessary. … I think vaccines in that respect are a sort of emergency measure to cure the symptoms of a society that hasn’t evolved and hasn’t developed in an ecologically grounded way. So, in that respect I’m against vaccines. But I mean, I realize the necessity of them, but I wish we would, if they’re that important for human beings to have a vaccine, we should trial them on ourselves and not on animals first. (CQI-0493)

In short, this participant expressed *distrust* in modern society and institutions (which they viewed as responsible for both pandemics and animal testing), yet this distrust also motivated their involvement in the clinical trial.

## Co-producing Experimental Subjectivities?

We now explore how participants talked about their own role as experimental subjects in relation to those of animals, particularly with respect to safety, care, differences between human and animal bodies, and consent.

### Reassurance and Concern

For some participants, the role of animal testing in vaccine development was not something they had given much thought, despite this being covered in the consent process: “I don’t know. I hadn’t really thought about it” (CQI-0347). Others, however, expressed a desire for more information (e.g., one wanted more information on the animal testing process, as part of having “more science” about the trial [CQI-0431]) or indicated that they had given considerable thought to animal testing. For these participants, animal testing served as a source of both concern and reassurance. In terms of concerns, six interviewees spoke about the potential increased risk of severe disease following vaccination, highlighted in animal trials against SARS, which presented risks that they needed to come to terms with. One interviewee explained that they “read the information sheet quite carefully,” and the information about the SARS animal trials “was slightly concerning, but not a massive concern. I thought okay, that’s an element there and you have to accept that” (CQI-0479).

For other participants, the knowledge that the vaccine had been tested on animals, and the results from animal testing, reduced concern, since “knowing that it’s been tested on animals as well is reassuring” (CQI-0363). As another participant put it:

I don’t think anyone would actually be willing to do human trials if there was no evidence to back up that you would be safe from it. I think most people would prefer it to be tested on an animal before. (CQI-0408)

Two participants, in articulating this idea, specifically referred to primate testing, with one stating that “no safety data at all from primates or other animal studies would be high risk, I would say” (CQI-0355). The second participant, who identified as a scientist and used the laboratory-specific term “NHP” (nonhuman primate), concluded, “I was pleased that it’d gone into NHPs and that had been assessed” (CQI-0346).

Thus, animal testing was perceived as a tool of public reassurance, enabling the first human test subjects to participate with some degree of confidence, because (as one put it) “it’s not just you that is just being tested” (CQI-0363). Animal testing was therefore perceived as playing an important role in allowing clinical trial participants to take risks in a responsible way (see also discussion of chains of care below). This reassurance is derived simultaneously from the scientists (who are trusted to have correctly conducted animal tests), and from the animals’ bodies, which are trusted (particularly those of fellow primates) to act as reasonable proxies for our own.

On the other hand, the use of primates specifically was a cause for concern for two participants, one being the participant who argued that modern society produces pandemics. For the other, the use of a highly intelligent animal was a source of discomfort, though they also did not see an alternative. The understanding that the vaccine had been tested on primates was a particular source of reassurance and concern, which came across in how frequently chimpanzees, monkeys, and primates were mentioned (forty-seven times by twenty-seven participants), despite chimpanzees not being used in any animal studies during the development of the Oxford vaccine. Indeed, chimpanzees cannot legally be used in medical testing in the UK and EU. The specific monkey species used in preclinical studies (rhesus macaques) was not referred to by any of our respondents, nor were the other species used (pigs and ferrets). The only exception to this was a reference to mice when a participant mentioned their knowledge of preclinical animal trials.

### Trial Speed

**Figure 2.**
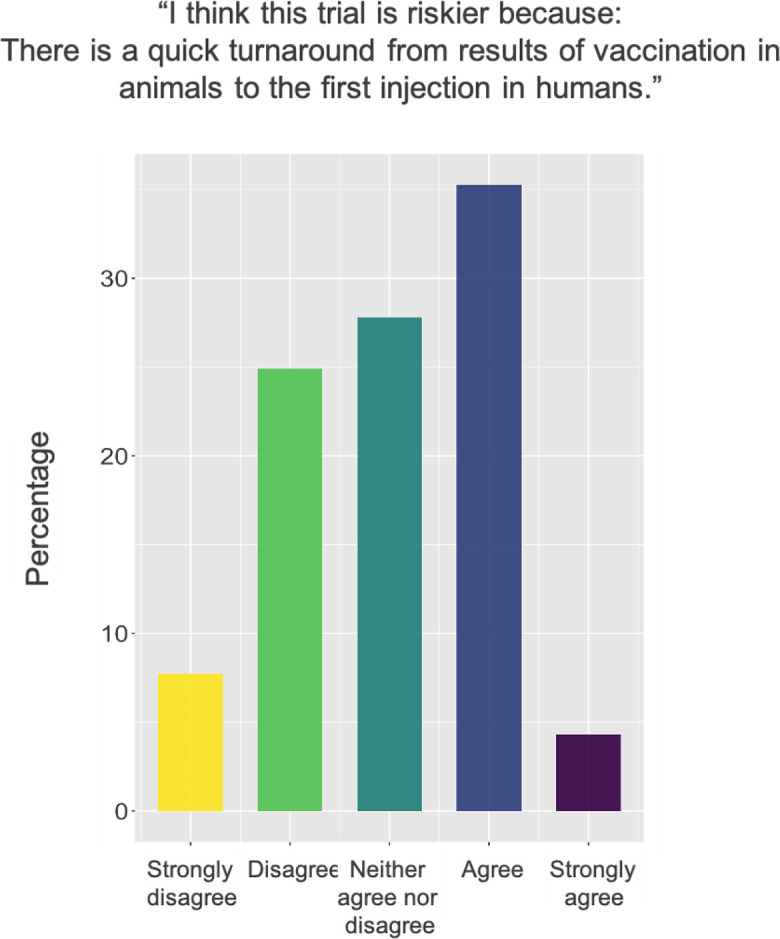
Survey responses to a question about the risk posed by the quick turnaround from animal to human testing.

A subject of particular public concern on COVID-19 vaccines was the speed at which human testing progressed. Questions on whether sufficient animal testing had been conducted circulated readily, despite indications from government regulators in various countries early in the pandemic that they would still require animal testing. Given the attention paid to this subject, we asked survey respondents whether they thought the trial was riskier because testing had progressed to human participants quickly after animal testing. Answers to this question displayed in Figure 2 indicate mixed views.

Respondents indicated awareness that the animal trial may have been quicker or less extensive than usual: “the animal studies were probably shortened slightly” (CQI-0481); “maybe [typically] they would do more extensive animal testing than they did” (CQI-0438); “It has been animal tested, but has been rushed through basically” (CQI-0375). One participant mentioned that this fast pace became a minor concern once they personally experienced side-effects:

I did get a little scared once I experienced the side-effects after having the initial vaccine because I was part of phase one, so once again, only six chimpanzees^1^ had had it before I did. And that’s not the best review in the world, but hey ho. But no. Yes, I wasn’t too worried. (CQI-0443)

For other participants, concerns about the fast pace of animal trials were mitigated by trust in the scientists: “my guess is that nothing that was important was missed” (CQI-0438), “the most important factor is the strong belief in the experts that it’s safe” (CQI-0432), and “they wouldn’t have asked for people to volunteer to have it if they hadn’t done preliminary checks” (CQI-0481).

Speed was also at times portrayed in a positive light, for example, demonstrating good organization:

I found it amazing how quick everything had been put into place and how well they’d done it, lining up all the approvals processes so that we were screened before they’d even finished the animal studies, and that was a remarkable thing. (CQI-0455)

Another participant who has worked on clinical trials as a scientist felt assured that speed and safety had been properly balanced: “this is as fast as we could go, safely” (CQI-0452). Others also cited the idea that in an outbreak scenario, speed is desirable. As one put it, “if we are trying to speed things up and move it forward, in a race almost against the virus that’s out there, you have to do that [move quickly] at some stage” (CQI-0481). Another even suggested that this ought to become a model for vaccine development in future outbreaks: “I think it should be done for a lot more vaccines, to especially help pandemics or epidemics” (CQI-0452).

### Chains of Care

Participants often spoke of their willingness to be involved in the trial in connection with their daily lives and responsibilities. Their willingness to be exposed to the risks of a vaccine trial for the wider social good was balanced against other kinds of personal commitments and obligations:

I personally would not have done it [participated in the clinical trial] if there hadn’t been animal trials. Because like I say, there are people who rely on me to do certain things for the continuation of their life. And the same thing if you had kids, you wouldn’t do it. (CQI-0334)

For this participant, animal testing is not merely a form of reassurance for the public *in general*, but specifically in the context of their own life. Human and animal test subjects are here envisioned as connected via a chain of care, in which animals care for human clinical trial participants by reducing their risk, enabling them to in turn provide care for others. There is a personalization of harm–benefit analysis in how to balance benefits to society against the risk of harm to individuals and therefore dependents. These reflections demonstrate how harm–benefit analysis cuts across scales and relations.

At the same time, people sometimes spoke about their own suitability as test subjects, citing confidence in their own robust immune systems as a reason for participating. People therefore spoke not only of the suitability of animals as the first experimental subjects but also about their own bodies as appropriate test sites prior to public distribution of the vaccine. In a sense, they saw animal test subjects as reducing the risk for them and their fellow clinical trial participants, and their own bodies as in turn protecting other humans with less robust immune systems. This idea was reinforced by some participants’ references to knowing people who had died of COVID-19, and their desire to prevent this from happening to others. In this vein, one participant described their role as one important step in the process of testing, which moves from mice, to monkeys, to humans:

my thought was somebody has to step up at the initial stage and test it. If it’s been tested in a laboratory, say, on mice and monkeys or other primates and that, there has to come a time when you have to trial it in human beings because that’s where it’s going to end up. (CQI-0481)

### Ideal Test Subjects

Seven interviewees spoke about themselves, and others, as being ideal test subjects through comparisons with guinea pigs. Guinea pigs feature as a common metaphor among clinical trial participants given their associations with experimentation (Abadie 2010; Fisher 2020). Using this metaphor, interviewees often spoke of a sense of their personhood being stripped away to serve a certain function for the trial. However, this was not meant negatively, as in some other contexts, for example, where the term is understood to connote dehumanization and stigma of trial participants (Fisher 2020). Rather, participants spoke of being a guinea pig as a form of service that they could perform for the greater good, as a “fit and healthy … young person who didn’t really have anything else to contribute to the pandemic” (CQI-0306). Two participants referenced seeing people such as doctors and virologists directly contribute, and explained that given their own lack of relevant expertise, being a guinea pig was a way “to do my bit” (CQI-0379).

Yet the guinea pig reference was also used to indicate risk, with one participant explaining that given vaccines are untested “there’s still a risk involved because you’re asking people to essentially be guinea pigs” (CQI-0363). Another noted that their family “thought it was very brave to be a guinea pig in the trial, which I hadn’t really thought was the case” (CQI-0385). One participant alternatively referred to themselves as being a “lab rat”: when asked about receiving information from the trial team the participant noted they would like to have “inside information” but understood “I’m strictly a lab rat for this” (CQI-0440). This usage hints at a slight differentiation between guinea pigs being used for experimentation and lab rats to produce data.

The use of animal metaphors, and view of humans as a (later) link in the chain of care that begins with research animals, indicates a willingness to view animals as the most suitable initial test subjects. However, one participant stood out in detailing a slightly different perspective on the appropriateness of certain bodies for use in experiments. Noting that they would prefer to live in a world where “there would be computer modelling and then human trials” rather than animal testing and that the primate testing “kind of bothered me,” they explained that a central reason for participating in the trial was to “put my money where my mouth was:” “if I want to live in a world where there’s no animal testing then I have to be prepared to put myself forward” (CQI-0479). For them, participating was acknowledging a solidarity with animals and also contributing to the possible end of animal trials in future. Their own body and those of fellow humans were therefore viewed as more ethical test sites than animal bodies. Yet they expressed a degree of hesitancy in committing to whether they would have participated had no animal testing been conducted – “I think would have still done it, hopefully” – suggesting that while they may disapprove of animal testing, they nonetheless do gain some reassurance from it. For them, animal bodies may not be ethically appropriate test sites, but they perhaps do still provide valuable information.

### Rights and Consent

A final connection made between animal and human test subjects related to agency and consent. Two participants noted the lack of choice that research animals have in whether to act as test subjects. Yet both still argued for the necessity of animal testing, despite its somewhat coercive nature:

It’s just difficult because we as humans can consent to being on a trial. Animals don’t have the same rights. I can’t see what the alternative would be. (CQI-0363)

In contrast, the second participant referred to their own willingness to participate, signaling that human testing is different because “humans can make the free will decision to do it” (and they personally would not have participated without animal testing; CQI-0334). Enabling human choice to participate, in this participant’s eyes, requires ignoring animals’ choices. However, animal testing also arguably provides more information for humans to make informed choices.

This emphasis on informed choice was echoed by another participant, who objected to the common guinea pig metaphor on the grounds that they (presumably unlike the guinea pig) consented fully to their involvement in research:

There was a report in…. It was either the BBC or the Guardian that used the word guinea pigs, which obviously is very common. …[I]t’s not a fair way of describing participants because informed consent is such a massive deal, and we go to such huge lengths to make sure that participants are well informed and understand their rights to withdraw, and all that sort of thing. (CQI-0347).

In this view, animal and human test subjects are quite distinct from one another, making comparisons via metaphors inappropriate.

## Conclusions

To conclude, we reflect on what asking clinical trial participants about their fellow nonhuman experimental subjects can tell us about: (i) public opinion on animal research; (ii) the co-production of human and animal experimental subjects; (iii) how vaccine and medicine testing does, or should, change in an outbreak; and (iv) what public involvement can offer to science.

### Public Opinion

Too often, discussions about animal research are highly polarized, with both “sides” of the debate seeking to characterize “the public” as agreeing with them (Davies et al. 2020; Hobson-West 2010). Yet, our results suggest considerable nuance to public views. We found broad support among clinical trial participants for the ethics embedded in UK animal research regulations, though they were often unaware of the standards used. This finding provides some reassurance that although animal research law is created through a messy and indirect process, whereby policymakers implement their understandings of what they think the public thinks (Hobson-West and Davies 2018), the views of this particular public are broadly reflected in law. Participants typically articulated the idea that animal testing is a “necessary evil” for developing medicines aimed at saving human lives, but still as something that made them uncomfortable and which would be unjustified for frivolous purposes such as cosmetic production. Animal research was therefore not viewed as justifiable under all circumstances but was dependent on the goal, methods, and quality of the science.

Despite its illegality in the EU and UK, cosmetic testing continues to loom large in the public imaginary, suggesting that even in this well-educated, pro-science public, substantial misconceptions about animal research persist. The frequent references to testing on chimpanzees (which were not used in testing the vaccine and cannot legally be used for invasive research in the UK and EU) echo this idea. Yet, whether these lingering misconceptions present any substantial problem for science is debatable, since they did not prevent people from volunteering as trial participants. Instead, these misconceptions tended to arise in contexts where participants articulated their nuanced positions about the circumstances under which animal testing is justified, and the troubling yet reassuring use of highly intelligent, human-like model organisms. Misinformation was, therefore, in this context not necessarily an impediment to trust or involvement in science (O’Connor and Weatherall 2019).

### Co-production

One key point around co-production was participants’ view of animals (in general, and specific species) as the most suitable initial test subjects. The commonly held view that animal testing is reassuring, providing confidence in the safety of the vaccine, suggests a certain degree of faith in the model organism concept whereby animals are assumed to be reasonable proxies for humans, despite the known shortcomings of this assumption (Davies 2010).

Participants also expressed an overwhelmingly positive view of their own roles as test subjects, using guinea pig and lab rat metaphors (which carried slightly different connotations) to express their sense of contributing to society and protecting the vulnerable during a health crisis. Comparing oneself to an animal was in this context not intended to convey dehumanization (Fisher 2020) but rather to express pride in one’s place in a chain of care. Volunteering for a clinical trial was thus tied up with notions of civic responsibility, and the obligations of the young and healthy to protect the vulnerable, which were prevalent in popular discourses during the COVID-19 pandemic (French Bourgeois, Harell, and Stephenson 2020).

We might expect that animals used in vaccine testing would be similarly viewed as model citizens who use their bodies to protect vulnerable humans. However, participants saw an important difference between themselves and research animals as being animals’ lack of choice. This is not to say that participants believed animals cannot choose, but simply that they viewed animals as coerced in the research laboratory. Recent STS scholarship has highlighted how animals in the laboratory can still exert agency and shape the direction and results of research (Despret 2004; Greenhough and Roe 2011). Yet the perception among clinical trial participants was that research animals do not truly “volunteer” (see Palmer et al. forthcoming). Perhaps, then, research animals were not viewed as model citizens, but more comparable to unruly individuals who would not choose to do the right thing and must therefore be coerced into protecting others.

### Testing in an Outbreak

Participants did, however, view themselves as having freely chosen to participate in the vaccine trial and hence as fundamentally different to research animals. This view stands in contrast to the view commonly expressed by healthy phase I clinical trial participants in the UK in nonoutbreak contexts that they have “no choice” but to enroll (Mwale 2017, 73). One potential reason for this is that during an outbreak, it would be inaccurate to say that healthy volunteers get nothing personally from their involvement. In the COVID-19 outbreak, everyone was at risk of contracting the virus (though some were at much higher risk of serious illness than others) and affected by nonpharmaceutical interventions such as lockdowns. In a sense, COVID-19 and its response could be viewed as “public experiments,” implicating everyone in society rather than being confined to a small group or a laboratory (Jasanoff 2006). Thus, by helping to develop an effective vaccine, participants could help minimize bereavement and disruption in their own lives as well as potentially protecting themselves from the virus.

Thus, in an outbreak where the general public is affected by an illness and responses to it, there is a resulting change to the motivations and demographics of phase I clinical trial participants. There is also a change to perceptions of the requirements for preclinical animal research, with many participants viewing the shortened time frame of testing as understandable and even desirable. This view echoes discussions around what, if anything, can be learned from the rapid development of COVID-19 vaccines for other diseases, including the possibility that the experience could “prompt a regulatory rethink” around testing (Ball 2020). For example, current regulations require animal testing before human trials to provide evidence of not only safety but also ideally efficacy; however, it is arguable what proof of clinical effect should be required under emergency conditions.

### Involving Publics in Science

Because trust in vaccines is ordinarily secured in part through large-scale testing over extended periods, relaxing efficacy requirements would require greater reliance on trust in scientists and scientific processes. As our data suggest, even in this case where animal testing was completed but the time frame was condensed, trust in science was important for participants’ willingness to be involved. Many pointed out that they are not themselves experts and had therefore placed their trust in the researchers. Participants’ frequent reminders that they are not experts suggests that they did not feel empowered to contribute technical knowledge in this context and that having experts guide them was important. This point resonates with a critique of “mode 2” science: that publics may prefer that experts take on leadership roles, and are frustrated by the “withholding [of] expertise” (Krzywoszynska et al. 2018, 802).

What our study participants did think they could contribute was not only their bodies as suitable test sites but also their perspectives on when, and why, animal testing is justified. Their reflections demonstrate how they might contribute to steering the direction and ethics of animal research through their personal beliefs and experiences, as a supplement to the more broad-brush picture presented by opinion polls (Ipsos MORI 2018) or policymakers’ preconceptions about “societal sentience” (Hobson-West and Davies 2018). As research on patient involvement in biomedical research has highlighted, there is an important role for publics in steering the direction, ethics, and approach of animal research (Gorman and Davies 2020).

## Supplemental Material

Supplemental Material, sj-docx-1-sth-10.1177_01622439211057084 - Co-producing Human and Animal Experimental Subjects: Exploring the Views of UK COVID-19 Vaccine Trial Participants on Animal Testing Click here for additional data file. Supplemental Material, sj-docx-1-sth-10.1177_01622439211057084 for Co-producing Human and Animal Experimental Subjects: Exploring the Views of UK COVID-19 Vaccine Trial Participants on Animal Testing by Samantha Vanderslott, Alexandra Palmer, Tonia Thomas, Beth Greenhough, Arabella Stuart, John A. Henry, Marcus English, Rebecca de Water Naude, Maia Patrick-Smith, Naomi Douglas, Maria Moore, Susanne H. Hodgson, Katherine R. W. Emary and Andrew J. Pollard in Science, Technology, & Human Values

Supplemental Material, sj-docx-2-sth-10.1177_01622439211057084 - Co-producing Human and Animal Experimental Subjects: Exploring the Views of UK COVID-19 Vaccine Trial Participants on Animal Testing Click here for additional data file. Supplemental Material, sj-docx-2-sth-10.1177_01622439211057084 for Co-producing Human and Animal Experimental Subjects: Exploring the Views of UK COVID-19 Vaccine Trial Participants on Animal Testing by Samantha Vanderslott, Alexandra Palmer, Tonia Thomas, Beth Greenhough, Arabella Stuart, John A. Henry, Marcus English, Rebecca de Water Naude, Maia Patrick-Smith, Naomi Douglas, Maria Moore, Susanne H. Hodgson, Katherine R. W. Emary and Andrew J. Pollard in Science, Technology, & Human Values

Supplemental Material, sj-docx-3-sth-10.1177_01622439211057084 - Co-producing Human and Animal Experimental Subjects: Exploring the Views of UK COVID-19 Vaccine Trial Participants on Animal Testing Click here for additional data file. Supplemental Material, sj-docx-3-sth-10.1177_01622439211057084 for Co-producing Human and Animal Experimental Subjects: Exploring the Views of UK COVID-19 Vaccine Trial Participants on Animal Testing by Samantha Vanderslott, Alexandra Palmer, Tonia Thomas, Beth Greenhough, Arabella Stuart, John A. Henry, Marcus English, Rebecca de Water Naude, Maia Patrick-Smith, Naomi Douglas, Maria Moore, Susanne H. Hodgson, Katherine R. W. Emary and Andrew J. Pollard in Science, Technology, & Human Values

## Note

1. The participant mistakenly refers to chimpanzees rather than rhesus macaques. No chimpanzees were used in testing the vaccine.
