# Supplementary material for: Co-producing Human and Animal Experimental Subjects: Exploring the Views of UK COVID-19 Vaccine Trial Participants on Animal Testing
Source: Sci Technol Human Values. 2021 Nov 15;48(4):909–37. doi: 10.1177/01622439211057084 (PMC10387720; doi:10.1177/01622439211057084)
Supplement: Supplemental Material, sj-docx-1-sth-10.1177_01622439211057084 - Co-producing Human and Animal Experimental Subjects: Exploring the Views of UK COVID-19 Vaccine Trial Participants on Animal Testing [file sj-docx-1-sth-10.1177_01622439211057084.docx]

**INTERVIEW GUIDE**

**Oral Information Giving for Semi-Structured Interviews**

This project has been reviewed by, and received ethics clearance through, the [ethics committee, reference].

Ensure the environment in which the meeting takes place is conducive to maintaining the privacy of the participant during the interview.

Ensure that verbal consent is taken.

Record using a digital recorder (if the participant has consented to this).

**Interview**

***Introduction****:*

Hello [x], my name is [x] from the [institution] COVID-19 Qualitative Study team.

Let me explain what will happen during this interview, once we start the recording I will read you a short paragraph about this study. Then I will ask you to confirm that you have read the information sheet and are happy to take part. I will also introduce the interview with you using your interview participant number.

I will just highlight a few key points about the interview.

- I will be recording the audio from our interview onto a digital recorder and the recording will be stored securely using your study number.
- If you would like to end the study at any point do let me know.

Are you happy for me to start recording? [Await confirmation]

<START RECORDING>

This is an interview with participant number: CQIXXX on DD/MMM/YYYY

Many thanks for agreeing to speak with me today. The [research group] is investigating public attitudes towards participation in vaccine trials. We hope this separate study will help better understand trial participation and inform future trials and engagement about vaccines. Participation in this qualitative study is voluntary and the responses you give won’t affect your involvement in the COV001 Vaccine Trial.

To allow us to continue can I confirm that you have read the interview participant information sheet. [Await confirmation].

***Questions/concerns***

Ask if they understand this before continuing and if they have any questions.

Can I confirm you consent to take part? [Await confirmation]. Ok, thank you.

Please could you tell me how old you are and how would you describe your gender?

(Please read the questions well beforehand and use them as a guide to structure the conversation. There is no need to read the questions word-for-word).

1. Views and experiences of COVID-19

- How has your experience of the COVID-19 pandemic been? - What have been the positive or negative aspects of the pandemic?

- When did you realise COVID-19 was a serious health issue?

- Have you been personally affected by COVID-19? Do you know anyone who has been ill with COVID-19?

2. Motivations

- What did you see as the advantages of volunteering for the COVID-19 vaccine trial?

- What are the disadvantages of volunteering for this trial?

- What improvements could be made about how the trial is performed?

3. Friends and family

- Did you ask anyone’s opinion before taking part in the trial? Is there anyone else that you know taking part in the trial? Who did you tell about your involvement in the trial?

- Did your friends and family approve or disapprove of you taking part in the trial? Did you have any unanticipated responses from those you told of your involvement?

- What were their concerns or objections (if any)? What were their approvals or encouragements (if any)?

- What do you think the public perception of the trial is? Have you been communicating about the trial to others? Have you shared your participation on social media? If yes, have you received approving or disapproving responses?

(These answers may be affected by the social media guidance, as they’ve been asked not to engage, so might be less willing to admit)

4. Vaccine outcomes (concentrate on this vaccine)

- Do you think this vaccine will work? What were your thoughts before and after the publication of the initial findings?

- Do you think you have received the COVID-19 vaccine?

- Since being involved in the vaccine trial, have you noticed any changes in your behaviour? (e.g. increased/decreased social distancing)

- If the vaccine is not effective will the trial still have been useful?

- Would you agree to be intentionally infected after receiving a COVID-19 vaccine to test if it works (this is called a human challenge or controlled human infection model study)?

- If the vaccine is shown to be safe and effective vaccine, how do you think it should be deployed? If a vaccine is shown to work, do you think health workers, key workers, and ‘at risk’ groups should receive the vaccine first?

- If this vaccine is shown to work, do you think people in the UK should receive a vaccine first? What if it was made in the UK?

- Are you aware of other vaccine trials? If yes please provide examples.

5. Trial experience during pandemic

- What has your experience been taking part in this trial during lockdown? Have there been any particular constraints or benefits? What would be different if there was not a lockdown?

- Do you think trials should be conducted faster during outbreaks?

- Which parts of a trial do you think can be speeded up (animal testing, human trial, regulatory approval, ethics review, manufacturing)?

- Do you think a vaccine should be manufactured even if there is a chance it does not work?

6. Risk/Safety

- When you decided to take part in the trial, how risky did you think it was? What is an acceptable level of risk for taking part in a vaccine trial?

- Is animal testing necessary to develop vaccines and treatments?

- If you experience any adverse events what will you expect will happen?

- Do you think some people should not take part in this trial? For what reasons?

- What risk(s) did you think about/worried you most before you took part in the trial?

- Have you been worried that you could become seriously ill as a result of your participation in the study?

- Were you surprised that if you developed Covid-19 disease during the trial you would not be entitled to additional healthcare (ie beyond the NHS)?

- Do you think vaccines developed during outbreaks are riskier? Do you think new vaccines are riskier than ones that have been around for a while?

7. Financial incentive

- Was the financial compensation a motivation for taking part in this trial?

- Do you think the financial compensation should be: none, lower, the same, higher. Do you think the financial compensation is fair? What amounts would be too little or too high?

- Would you have taken part in this trial without any compensation?

8. Attitudes to vaccines

- Have you been fully vaccinated? And if you have children, have they been fully vaccinated? Are you supportive of all vaccination? Why do you think some people oppose vaccination?

- Do you know people who have been opposed to the vaccine or who you think would be?

- Do you agree with mandatory vaccination? Should a COVID-19 vaccine be made mandatory?

9. Before this study, what was your knowledge of clinical trials? (heard, or knew about them)

Have you been in a clinical trial before?

If yes - How many and what kind of trial?

Was that a positive experience?

10. Would you take part in other clinical trials?

- Yes – why?

- No – why?

- Don’t know – [prompt: for example, a similar vaccine trial, a trial for other medicines, or this was a one-off, open to possibility]

- What would encourage you to take part in another trial? What would discourage you from taking part in another?

11. Media representation

- How do you think this trial has been represented in the media? Have you seen other trial participants in the media? What did you think?

- Have you been approached by the media during this trial? Have you engaged with the media? Do you intend to engage with the media? What has your experience of the media been?

- Have you been communicating about the trial on social media?

- Were you aware of any 'fake news' or misinformation surrounding the trial?

(May need to reassure that all answers are confidential and there is no right/wrong answer)

12. Trial progress/feedback

- Could anything be improved about how the trial is performed so far?

- What has been the best aspect of being in the trial? What has been the worse/most cumbersome aspect of being in the trial?

13. Conclusion

Is there anything else that you would like to comment on that I haven’t already asked you about? Or any questions about this study?

Thank you very much for your time and the information you shared today.

<STOP RECORDING>
