# Supplementary figures and images for: Co-producing Human and Animal Experimental Subjects: Exploring the Views of UK COVID-19 Vaccine Trial Participants on Animal Testing
Source: Sci Technol Human Values. 2021 Nov 15;48(4):909–37. doi: 10.1177/01622439211057084 (PMC10387720; doi:10.1177/01622439211057084)

**Survey demographics**

# **
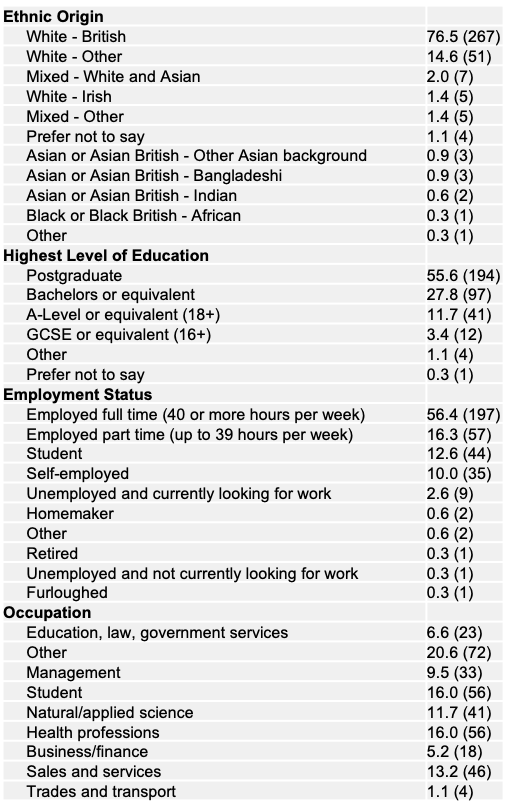

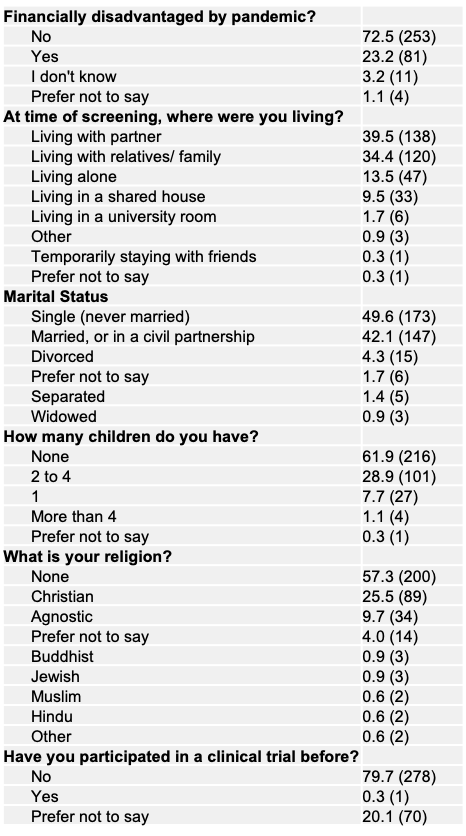

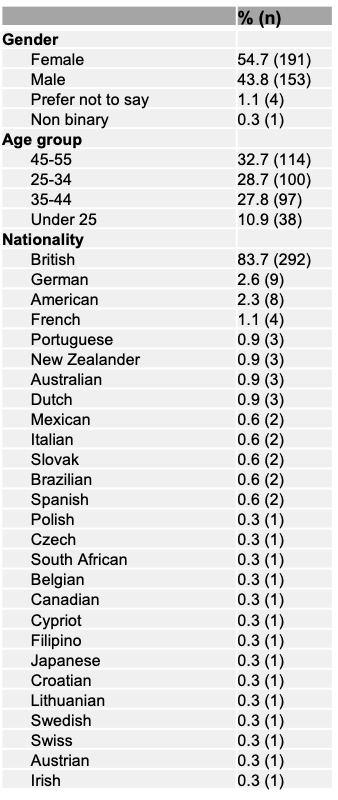
**

Supplement: Supplemental Material, sj-docx-2-sth-10.1177_01622439211057084 - Co-producing Human and Animal Experimental Subjects: Exploring the Views of UK COVID-19 Vaccine Trial Participants on Animal Testing [file sj-docx-2-sth-10.1177_01622439211057084.docx]
